# Estimation of bacterial diversity using next generation sequencing of 16S rDNA: a comparison of different workflows

**DOI:** 10.1186/1471-2105-12-473

**Published:** 2011-12-14

**Authors:** Jorge Barriuso, Jose R Valverde, Rafael P Mellado

**Affiliations:** 1 Centro Nacional de Biotecnología (CSIC), c/Darwin 3, 28049 Madrid, Spain

## Abstract

**Background:**

Next generation sequencing (NGS) enables a more comprehensive analysis of bacterial diversity from complex environmental samples. NGS data can be analysed using a variety of workflows. We test several simple and complex workflows, including frequently used as well as recently published tools, and report on their respective accuracy and efficiency under various conditions covering different sequence lengths, number of sequences and real world experimental data from rhizobacterial populations of glyphosate-tolerant maize treated or untreated with two different herbicides representative of differential diversity studies.

**Results:**

Alignment and distance calculations affect OTU estimations, and multiple sequence alignment exerts a major impact on the computational time needed. Generally speaking, most of the analyses produced consistent results that may be used to assess differential diversity changes, however, dataset characteristics dictate which workflow should be preferred in each case.

**Conclusions:**

When estimating bacterial diversity, ESPRIT as well as the web-based workflow, RDP pyrosequencing pipeline, produced good results in all circumstances, however, its computational requirements can make method-combination workflows more attractive, depending on sequence variability, number and length.

## Background

The application of nucleic acid-based techniques is a useful tool for diversity studies in natural habitats [1] and a number of culture-independent nucleic acid-based methods have been used to characterise microbial communities. Next Generation Sequencing (NGS) of hypervariable regions from small-subunit ribosomal RNA genes is a conventional tool to analyse the composition and diversity of microbial communities in several habitats [2-4]. NGS allows gene sequencing from complex environmental samples [2,5,6] favouring the analysis of bacterial diversity in a comprehensive manner [7].

Taxonomy-independent studies are used to analyse diversity at different similarity levels [8-12]. Several analytical methods included in different software packages are available for these processes [9,12-22].

Typical diversity data analysis workflows start by assessing data quality and removing primers and noise. This is usually followed by a multiple sequence alignment (MSA) used for distance calculation, which is the basis for clustering sequences into Operational Taxonomic Units (OTUs) at the desired dissimilarity, usually 3% for species and 5% for genera [2,20]. Additional filtering steps may be inserted to remove redundant gaps, even sequence ends, and detect repeated or closely related sequences to reduce the amount of data to be processed [20-26]. Filtering processes are also used to improve sequence quality [24-26]. Each step can be carried out using a variety of tools, and different tool combinations are commonly used to tailor the analysis to the original data [e. g. 26]. Some approaches avoid MSA by using pairwise alignments to compute distances [20,22,23].

Observed OTU counts and relative abundances are representative of actual diversity, yet we cannot be sure that total diversity has been identified unless an appropriate sample size has been employed, which depends on diversity and hence is difficult to predict. For this reason estimates of species richness must be considered, such as rarefaction curves and ACE or Chao1 estimators, among others.

Comparative studies of diversity in environmental samples are usually carried out either by comparing the above estimators or using phylogenetic information, as implemented in UniFrac [27]. Lately, approaches to derive OTU numbers from taxonomic classifications produced by the RDP classifier [28] have been proposed, however, this approach is limited by the existing data in the databases [29].

Recent reports have compared some of the methods available and their potential advantages [11,25,30] computing OTU counts, however, there is still little knowledge on how these combinations affect workflow performance under different conditions, and the specific suitability for differential diversity studies. In order to acquire useful rules and advice on the choice of workflow, we employed the most commonly used tool combinations to generate the corresponding workflows.

Application of these techniques has been greatly facilitated by the availability of tool collections packaged for easy setup and use, such as QIIME [31], which includes many of the tools analysed here, allowing scientists to select and combine specific tools to suit their needs, highlighting the requirement for studies to compare the relative merits of each method combination workflow.

We tested three different alignment strategies: *ab initio *alignments using the progressive alignments of MUSCLE [14], the MAFFT partition tree method [16], and reference guided alignments as implemented in Mothur [19]. The effectiveness of filtering and pre-clustering was tested on Mothur alignments. We checked the effect of alternative distance calculation approaches using the Jukes-Cantor correction for multiple nucleotide substitution, as implemented in DNADIST [2,12,32-34], the uncorrected distance with the gap count method from Mothur [19] (hereinafter referred to as "Mothur distance"), and the k-mer based distance method from MAFFT [16] (referred to as "MAFFT distance"). Finally, all combinations of MSA and distance matrices were clustered using Mothur [19].

In addition to these combinations, we also considered other popular streamlined OTU identification workflows: Mothur used as the only tool for the whole pipeline with and without pre-clustering, ESPRIT [20], which uses pairwise alignments to compute distances, the recently published CROP [23], which uses an unsupervised Bayesian clustering method, Otupipe, which is based on UCLAST/USEARCH [22], and the RDP pyrosequencing pipeline [21], which uses Infernal [35,36] alignments and its own complete linkage clustering method.

To compare these workflows, we needed to resort to well-defined datasets representative of typical experimental set ups: we used reference 16S sequence data of various lengths using synthetic data developed by us, as well as datasets proposed by Youssef [37], Quince [26], Hao [23,38] and Huse [24]. These sets test accuracy by using a single sample with a limited number of species. To analyse workflow suitability for differential diversity studies, we used real-world field data of hypervariable V6 16S rDNA sequences [32] retrieved at different times under different environmental conditions. The V6 region is a common choice for this kind of analysis as it has been proven to yield results similar than longer sequences [11,37], and is a sensible choice for short-length sequencing approaches.

In order to compare clustering results one needs to account for potential method-dependent variability due to randomness in the workflow (e.g. Mothur randomly selects the cluster to group in case of a tie and CROP uses a Markov Chain Monte Carlo -MCMC- approach). We have collected statistics on the variability results obtained with Mothur, ESPRIT and CROP.

## Results

### Reference set of short-length hypervariable V6 16S rDNA

The results obtained with short V6 region sequences using our synthetic data sets derived from 60 species are shown in Table 1, which indicates the number of OTUs obtained at 3%, 5% and 10% dissimilarity levels with each workflow. Manual checking of the selected sequences revealed that two of them were identical, (EU930421 and DQ989484), two pairs of sequences had a dissimilarity level below 5% (X90760 and AM743175, AF328681 and EU551675), and five pairs of sequences had a dissimilarity level below 10% (AM935529 and EU631792, AF114805 and AY894325, AY234615 and EF173341, AF114805 and AY894325, U855746 and EU360125). Therefore, the expected number of OTUs should be 59 at 3% dissimilarity, 57 at 5% dissimilarity, and 52 at 10% dissimilarity, which are precisely the results obtained with ESPRIT, Otupipe and the RDP pipeline. CROP correctly identified the number of OTUs at 3% when only 60 sequences were used, yet overestimated them at 5% and when 50 repeated copies of them were used. Mothur, on the other hand, underestimated the number at all dissimilarity levels, both with and without the pre-clustering step, due to the removal of sequence fragments during the reference-guided alignment step, converting divergent sequences into identical or more closely-related fragments (http://www.mothur.org/wiki/Align.seqs).

**Table 1:** Accuracy test

	60 seqs			50 × 60 seqs		
Reference expected values	59	57	52	59	57	52
CROP	59	59	59	60	60	60
ESPRIT	59	57	52	59	57	52
MAFFT+JC	58	56	50	58	57	51
MAFFT+MAFFT	59	59	59	59	59	59
MAFFT+Mothur	59	56	51	59	57	54
Mothur+JC	55	55	55	2992	2992	2992
Mothur+MAFFT	41	40	36	252	251	243
Mothur+Mothur	48	48	48	48	48	48
Mothur+PreC+JC	44	44	44	43	43	43
Mothur+PreC+MAFFT	47	47	47	47	47	47
Mothur+PreC+Mothur	48	48	48	48	48	48
MUSCLE+JC	58	56	50	63	59	59
MUSCLE+MAFFT	59	59	59	59	59	59
MUSCLE+Mothur	59	56	55	66	62	56
Otupipe	59	57	52	59	57	52
RDP	59	57	52	59	57	52

OTUs observed with each of the workflows analysed at distances of 3, 5 and 10% for the datasets containing 60 test sequences and 50 replicas of the same (50 × 60). JC stands for Jukes-Cantor and PreC for the pre-clustering step applied after Mothur MSA. Combined workflows are indicated stating first the method used for alignment (MAFFT, Mucle, Mothur or Mothur with pre-clustering), and then the distance method used (Jukes-Cantor, MAFFT or Mothur). Clustering was performed with Mothur for all combined workflows.

Regarding combined workflows, it is noticeable that both MAFFT and MUSCLE alignments produce good results, and that the combinations of Mothur alignments with either Jukes-Cantor corrected or MAFFT distance calculations give abnormally high counts with the 50 replica data set. The MAFFT distance calculation had trouble to discriminate between 3%, 5% or 10% dissimilarity levels (59 OTUs), in contrast to Mothur or Jukes-Cantor corrected distances.

### Reference sets of short length mutated V6 rDNA

To highlight the effect of sequence alignment on clustering and diversity estimation we mutated the 60 reference sequences and arranged them in two different groups: stacked or interspersed. Since random mutations have been added, we now expect diversity to be higher at 3% distance and conserved at 5% or more. Results are summarized in Table 2: CROP gives abnormally large counts, ESPRIT overestimates at 3% dissimilarity but returns to acceptable estimations at 5% and 10% distances. Mothur alignments exceedingly overestimate diversity on account of the mentioned abnormal alignments, with the preclustering step reducing OTU counts, which is likely because these discard many sequences as if they were sequencing errors. Once again MAFFT and MUSCLE alignments give better results when combined with Jukes-Cantor corrected or Mothur distances, but not with MAFFT distances.

**Table 2:** Alignment test

	50 × 60 mutated (interleaved)			50 × 60 mutated (stacked)		
	3%	5%	10%	3%	5%	10%
Reference expected values	59	57	52	59	57	52
CROP	1959	1954	1955	1865	1850	1850
ESPRIT	193	59	56	205	59	56
MAFFT+JC	141	101	85	132	113	113
MAFFT+MAFFT	2289	1708	80	2289	1777	1270
MAFFT+Mothur	261	119	96	279	121	121
Mothur+JC	2947	2947	2948	2985	2985	2985
Mothur+MAFFT	899	685	477	2999	2999	2999
Mothur+Mothur	1087	923	736	1100	930	888
Mothur+PreC+ JC	1198	1198	1198	1333	1333	1333
Mothur+PreC+MAFFT	1328	1328	1328	1346	1346	1346
Mothur+PreC+Mothur	1080	938	938	1087	940	915
MUSCLE+JC	70	59	44	2999	2999	2999
MUSCLE+MAFFT	2287	1707	80	2288	1785	1269
MUSCLE+Mothur	264	64	57	571	466	466
Otupipe	139	91	59	144	95	61
RDP	59	58	53	59	57	52

OTUs observed with each of the workflows analysed at distances of 3, 5 and 10% for the datasets containing 50 different mutated replicas of the 60 test sequences stacked or interleaved.

There are obvious differences in the calculations depending on sequence ordering. A strong influence arises from the alignment step, verified by looking at the MSA. We know that each sequence contains only one change and therefore, deviations are easily identifiable. In both cases (stacked or interspersed) MAFFT and MUSCLE produced shorter alignments than Mothur, with or without pre-clustering (400 MAFFT, 686 MUSCLE sites vs. 2641 Mothur sites for stacked mutants, and 445 MAFFT, 372 MUSCLE sites vs. 2553 and 2436 Mothur sites for interspersed mutants). Evening sequence ends in Mothur produced useless alignments. Strong differences can also be detected between both datasets, with the combination of MUSCLE and Jukes-Cantor yielding very high values with stacked data. ESPRIT is more resilient to sequence order, as it avoids the MSA step, showing only minor differences in results between datasets and consistently producing good Chao1 estimates (Additional file 1, Table S1.3). Otupipe, which also avoids the MSA step, displays a similar behaviour. Only RDP produced results consistent with the expected values and was able to avoid the influence of sequence order in the input data.

### Reference sets simulating 454 NGS data of 16S rDNA

Table 3 summarizes the results obtained when analysing the V5 region datasets from Quince et al. [26]. CROP, ESPRIT, Otupipe and the RDP pipeline results are in line with published data [23,26], with CROP and Otupipe giving low estimates. When Mothur was used to compute the alignment with only vertical filtering to remove gaps, extremely large OTU counts were obtained, independently of the distance algorithm used (data not shown). However, Mothur behaviour could be corrected by removing uneven ends in the filtering step ("trump"), producing estimates closer to those previously published but lower than ESPRIT. MUSCLE alignments were able to produce acceptable results with the Artificial but not the Priest Pot data. MAFFT alignments produced very large OTU counts. In all cases, the worst results were obtained when MAFFT k-mer distances were used.

**Table 3:** Quince's 454 data

	Artificial			Priest Pot		
	3%	5%	10%	3%	5%	10%
CROP	41	25	15	562	246	42
ESPRIT	248	77	38	1115	773	394
MAFFT+JC	686	686	686	3764	3764	3764
MAFFT+MAFFT	31933	31933	31933	15984	15984	15984
MAFFT+Mothur	1756	1756	1756	6672	6672	6672
Mothur+JC	49	36	33	640	537	537
Mothur+MAFFT	4276	4276	4276	2824	2824	2824
Mothur+Mothur	113	53	53	766	642	642
Mothur+PreC+ JC	61	40	36	662	482	482
Mothur+PreC+MAFFT	3864	3141	3141	1905	1625	1625
Mothur+PreC+Mothur	136	65	46	810	575	575
MUSCLE+JC	146	146	146	4059	4059	4059
MUSCLE+MAFFT	33491	33491	33491	15718	15718	15718
MUSCLE+Mothur	258	258	258	6433	6433	6433
Otupipe	66	39	24	793	570	302
RDP	250	94	43	1209	862	456

OTUs observed with each of the workflows analysed at distances of 3, 5 and 10% for Quince's Artificial and Priest Pot datasets. For the Priest Pot data, 855 OTUs at 3% and 699 at 5% were previously estimated by Quince et al. [26].

### Reference set using a large number of short length reads

Recent advances in pyrosequencing have increased the size of reads that can be collected, making this technology an attractive tool for biodiversity studies. To analyse the likely behaviour of these new approaches we have resorted to a well- known and established dataset consisting of a large number of sequences (340150) previously reported by Huse *et al*., [24]. Results are shown in Table 4: CROP failed to successfully deal with this large dataset. For the remaining approaches, the main problem arises from the size of the distance matrices generated, which can easily reach terabyte file sizes, becoming unmanageable for clustering programs such as Mothur. In these cases, the data can be processed either by including a preclustering step (e.g. in Mothur or combining MUSCLE with USEARCH), or analyzing unique sequences (albeit at the cost of losing information on abundance), or by obviating the need for an MSA (like ESPRIT or Otupipe). The RDP pipeline was able to build the alignment, but it could not be processed in the standard manner, and it did require specific RDP staff assistance to process this dataset.

**Table 4:** Huse short read data

	3%	5%	10%
CROP	NC	NC	NC
ESPRIT	6464	3308	1402
Unique:MAFFT+JC	23442	23442	23442
Unique: MAFFT+MAFFT	23445	23445	23445
Unique: MAFFT+Mothur	23445	23445	23445
Unique:Mothur+JC	23441	23441	23441
Unique: Mothur+MAFFT	23441	23441	23441
Unique: Mothur+Mothur	18210	18210	18210
Mothur+PreC+ JC	15594	15594	15594
Mothur+PreC+MAFFT	15601	15601	15601
Mothur+PreC+Mothur	14776	14776	14776
Unique:MUSCLE+JC	22816	22816	22816
Unique:MUSCLE+MAFFT	23444	23444	23444
Unique:MUSCLE+Mothur	21318	21318	21318
Otupipe	2149	1422	878
RDP	4228	2932	1777

OTUs observed with each of the workflows analysed at distances of 3, 5 and 10% for a large number of short length reads dataset [24]. NC = non computable.

None of the workflows involving an MSA followed by distance-matrix calculation and clustering could be completed due to the huge size of the distance-matrix. When the 23445 unique sequences in the dataset were used to reduce dataset size, with or without preclustering, all of them gave very high OTU counts and were unable to make distinctions at different similarity levels. Only ESPRIT, Otupipe and the RDP pipeline were able to produce different results at each dissimilarity level (Table 4).

### Reference sets using near-full length 16S rDNA sequences

We have analysed two near-full length sets with similar sequence counts: Hao's axillary skin reference data comprised low diversity, while Youssef's prairie soil samples contained higher diversity. The results are summarized in Table 5: CROP results show a strikingly high number of OTUs. In all cases fairly similar results were obtained with both datasets and Mothur gives acceptable figures either alone or in combined workflows, again with the exception of k-mer based distance calculation (MAFFT), which overestimates OTU numbers, albeit much less than the estimates obtained when shorter sequences were analysed.

**Table 5:** Near-full length sequences

	Skin axillary			Prairie soil		
	3%	5%	10%	3%	5%	10%
CROP	1009	1009	1009	1128	1128	1128
ESPRIT	59	43	26	504	340	162
MAFFT+JC	47	39	39	490	346	183
MAFFT+MAFFT	266	126	109	1007	895	714
MAFFT+Mothur	50	39	36	503	351	181
Mothur+JC	47	39	36	491	352	184
Mothur+MAFFT	269	126	109	977	872	689
Mothur+Mothur	54	40	38	535	396	204
Mothur+PreC+ JC	47	39	36	491	353	268
Mothur+PreC+MAFFT	287	127	109	977	877	836
Mothur+PreC+Mothur	55	40	38	536	397	305
MUSCLE+JC	50	39	37	480	337	175
MUSCLE+MAFFT	266	126	109	1007	895	714
MUSCLE+Mothur	58	40	38	487	339	173
Otupipe	49	37	26	490	336	160
RDP	60	43	27	504	348	175

OTUs observed with each of the workflows analysed at distances of 3, 5 and 10% for the skin axillary microbiome data described by Hao *et al*. [23], and tall grass prairie soil data [37].

### Experimental V6 16S rDNA sequences from soil samples

These datasets compare differential diversity estimations before and after herbicide treatment with a control: differences in diversity should be consistent across workflows. The results are shown in Table 6. CROP gives the lowest diversity estimates in all cases. In the remaining workflows, the untreated and glyphosate-treated soils in practically all cases are more diverse than the GTZ-treated soil when comparing sampling times. The actual figures varied considerably depending on the workflow used. Combined workflows showed again a tendency to produce higher OTU counts when MAFFT was used to calculate distances.

**Table 6:** Comparative diversity analysis

	Control		GTZ		Glyphosate	
	t1	t2	t1	t2	t1	t2
CROP	812	286	414	625	454	477
ESPRIT	1631	1053	1227	922	1951	1102
MAFFT+JC	1936	1087	1025	975	1977	1088
MAFFT+MAFFT	2792	1588	1971	1329	3296	1577
MAFFT+Mothur	2112	1215	1655	1163	2646	1326
Mothur+JC	3464	1622	3842	823	5024	1090
Mothur+MAFFT	2789	1589	1969	1328	3323	1574
Mothur+Mothur	1730	1128	1310	982	2122	1209
Mothur+PreC+JC	2479	1460	1958	826	3134	1498
Mothur+PreC+MAFFT	2471	1459	1961	1252	3135	1495
Mothur+PreC+Mothur	2063	1143	1890	1010	2516	1221
MUSCLE+JC	1762	1247	1257	950	2020	1238
MUSCLE+MAFFT	2790	1587	1970	1330	3296	1577
MUSCLE+Mothur	2278	1352	1800	1059	2772	1477
Otupipe	1314	881	938	795	1428	948
RDP	1762	1106	1236	901	1932	1094

OTUs observed with each of the workflows analysed. For simplicity, only results at 3% dissimilarity are shown from pooled samples collected at two different times from control, GTZ and glyphosate treated soils [32]. Full data at 3, 5 and 10% is provided in the comprehensive OTU results table of the Additional file 1, Tables S1.1, S1.2 and S1.3.

### Computational variability of results

Most calculations were repeated to confirm the observed results. This showed, in several instances, the same computation producing different results due to intrinsic algorithmic randomness. These expected variations were measured repeating the calculations 20 times with three selected datasets under the same conditions. The results obtained are presented in Table 7. ESPRIT did not show any variability, Mothur showed a slight variability in some instances, which should not change OTU estimates, and CROP showed a slightly higher variability, which may induce minor estimate changes.

**Table 7:** Observed output variability

		50 × 60 seqs mut stacked				50 × 60 seqs mut interleaved				Prairie soil			
	N	μ	Err	Min	Max	μ	Err	Min	Max	μ	Err	Min	Max
CROP 3%	20	1852.2	2.16	1847.6	1856.7	1954.6	0.83	1952.8	1956.3	1128	0	1128	1128
CROP 5%	20	1853.5	2.43	1848.4	1858.5	1952.4	0.66	1951	1953.7	1128	0	1128	1128
CROP 10%	20	1850.9	1.65	1847.4	1854.4	1951.7	0.62	1950.4	1953	1128	0	1128	1128
ESPRIT 3%	20	205	0	205	205	193	0	193	193	565	0	565	565
ESPRIT 5%	20	59	0	59	59	59	0	59	59	381	0	381	381
ESPRIT 10%	20	56	0	56	56	56	0	56	56	180	0	180	180
Mothur 3%	20	1465.3	0.23	1464.8	1465.8	1173	0	1173	1173	541	0	541	541
Mothur 5%	20	1465.3	0.23	1464.8	1465.8	1173	0	1173	1173	400	0	400	400
Mothur 10%	20	1465.3	0.23	1464.8	1465.8	1173	0	1173	1173	347	0	347	347
Mothur+PreC 3%	20	1490.6	0.16	1490.3	1490.9	1197.3	0.12	1197	1197.6	541	0	541	541
Mothur+PreC 5%	20	1490.6	0.16	1490.3	1490.9	1197.3	0.12	1197	1197.6	400	0	400	400
Mothur+PreC 10%	20	1490.6	0.16	1490.3	1490.9	1197.3	0.12	1197	1197.6	348	0	348	348

Observed output variability with 20 equal runs of CROP, ESPRIT and Mothur (with (PreC) and without preclustering). N is the number of observations for all samples; μ is the observed mean of the sample. Standard error (Err) and confidence intervals (Min and Max) were calculated from these values, as described in Methods. Many of the CROP calculations failed to complete in this test, as reflected by N (Prairie soil), and statistics were adjusted accordingly.

Since CROP estimates are obtained via independent runs for each distance, this may result in a seemingly contradictory output, where OTU counts at 5% might be greater than at 3% (e.g. one of the runs for the stacked mutants produced 1865 OTUs at 3%, and another 1869 at 5%).

## Discussion and Conclusions

We have analysed the most commonly used workflows applied for bacterial diversity studies including simple, one-tool, workflows and tool combinations, and compared them for accuracy, results variability and efficiency under a variety of conditions representative of Illumina-based, 454-based, near full-length and differential diversity studies.

While more complex workflows can be devised (e. g. using RDP web alignments or ESPRIT distance calculations or clustering as optional combinatorial steps), these are rarely reported except when evaluating new algorithms [23,26] and are inconvenient for routine use. The most relevant patterns from the wealth of information generated by our analysis are highlighted below.

### Sequence processing

The relevance of initial data quality to the reliability of the results has been repeatedly noted, leading to the recommendation of filtering raw data according to quality, removal of chimeras and experimental noise, and ensuring all sequences correspond to the same region of 16S rDNA. This last step usually relies on the selection of experimental primers, but may sometimes benefit from the additional removal of non-overlapping sequences ("trump" filtering in Mothur). This step may arguably remove important elements in an alignment (e. g. if terminal gaps are significant), but it was the only way to obtain sensible results with the datasets from Quince *et al*. [26]. However, its use with our artificially mutated sequences resulted in useless alignments, showing that this option must be applied with caution.

When a large number of short length sequences are used [24], it is easy to find many individuals with the same sequence. Identifying and using unique sequences exerts a major impact on reducing computation time, especially when dealing with modern vast datasets (in the hundreds of thousands or millions of sequences).

### Alignments

ESPRIT, which uses pairwise alignments, generally produces more accurate results than workflows relying on MSAs. Pairwise alignments; however, do not consider likely evolutionary relationships or structural properties, which is why MSA tools are often preferred. Alignments based on a reference alignment are shown to be very useful, while improving quality, as can already be seen from the RDP pipeline results; however, we observe that using MAFFT or MUSCLE to obtain *ab initio *MSAs gave better results than Mothur reference-guided alignments for short sequence lengths. This is most evident with our engineered mutated datasets, and is likely due to reference guided alignments selecting different reference sequences for related mutants, leading to an MSA where related sequences include more variability than actually exists. Hence, Mothur MSAs should always be filtered to remove redundant gaps if other tools are to be used for distance calculation [19]. MAFFT and MUSCLE frequently identify the mutation correctly and produce more accurate alignments, as can be seen by inspecting the aligned stacked dataset. This advantage is diluted as sequence length increases (datasets from Quince, Hao and Youssef), since the availability of more information increases the accuracy of reference identification.

Another major difference is related to the computational time required for constructing the alignments. Both MAFFT and MUSCLE have a well-defined behaviour: MAFFT time complexity is O(N^2^L) [39], N being the number of sequences and L their length, and MUSCLE uses multiple iterations with a cost of O(NL+L^2^) per iteration; however, ESPRIT, CROP and Mothur compute more expensive Needleman-Wunsch alignments (cost O(L_1_·L_2_), or O(L^2^) for equal-length sequences) and rely on a previous filtering step to reduce N, the number of sequences to be compared. ESPRIT and CROP require (N·(N-1)) comparisons to build the distance matrix; one may expect ESPRIT k-mer filtering or CROP unique identification step to become less efficient for longer, relatively distant sequences. In the case of Mothur only N comparisons -each sequence against its reference, selected using one of various filtering methods (k-mer, blastn and suffix trees)- are needed. Additional file 2, Table S2 summarises the computational time needed for each step in the diversity analysis.

Summarizing, ESPRIT works better for short and closely related sequence reads and is usually very fast; however, it is much slower when tens of thousands of sequences are considered. MUSCLE is preferred for short sequence lengths, but its long running time puts it at a disadvantage when sequences are long, deficiently grouped, or too many are used. Mothur generally achieves the fastest alignments when using a reference database, although there are concerns with the relative merits of this approach [19,35]. In the case of long sequences, MAFFT offers a very good balance between speed and quality.

Special mention must be made for MSAs of huge datasets in the order of hundreds of thousands of sequences: in these cases, MUSCLE is unable to directly align the data and requires a previous "clumping" and filtering step with USEARCH to guide the final alignment, or selection of unique sequences prior to alignment. In any case, subsequent processing makes it advisable to favour working with unique sequences or a pre-clustering step when available, both to reduce computation time and produce manageable datasets. Regarding computation time, this is the most expensive step, but only becomes a real issue with modern computer architectures when dealing with very large sequences (which is not usually the case in diversity studies) or huge numbers of sequences. In the last case, the selection of unique sequences and/or preclustering helps to reduce computation to acceptable times (from days or even months down to hours). In the case of MUSCLE it may additionally be required to reduce the number of iterations and increase the maximum memory used (--maxiter 2 --maxmb 10000) (Additional file 2, Table S2).

### Distance calculation

It has been argued that the direct calculation of distances from pairwise comparisons yields better distance matrices than distances calculated from MSAs, and indeed one expects less gaps in a pairwise than a multiple alignment, as reflected in the ESPRIT package, which tends to produce the lowest number of OTUs, in agreement with earlier findings [20,25]. However, the computational cost increases with the number of sequences, making it more attractive to use MSA-based methods for large datasets (Additional file 2, Table S2). We see that both Jukes-Cantor corrected distances and Mothur distances give consistent and sensible results. Mothur distances are inclined to produce lower diversity and MAFFT distances tend to give 30-35% overestimations in the number of OTUs. The behaviour of MAFFT distance calculation is explained by its k-mer-based approach, where few changes in short sequences will have a bigger impact on overall distance than similar changes in longer ones.

In conclusion, distances derived from pairwise alignments produce better results, but may become inconvenient for long or large numbers of sequences. In these cases, Mothur distances produce lower OTU counts than Jukes-Cantor. K-mer-based distances are not currently recommended.

It is worth noting here that distance matrices inherently grow in size with the number of sequences squared. This may become a problem with modern datasets consisting of hundreds of thousands or millions of sequences, where terabyte-sized matrices may be generated. Thus, methods that avoid generating distance matrices may be more advisable for this data.

New versions of other popular alignment methods tuned for NGS analysis are expected soon (Higgins, D, personal communication), and it will be interesting to test them as soon as they become available.

### Clustering analysis

We have compared the results obtained with different clustering strategies: CROP, ESPRIT, Mothur, USEARCH and RDP. To simplify the study, we did not consider other clustering tools such as CD-HIT-454, which uses heuristics that are too stringent for richness analysis [40]. It is difficult to compare the different clustering strategies as ESPRIT, CROP, USEARCH and RDP have only been considered in their own workflow, except for the fact that Mothur is the easiest to integrate in complex workflows and offers a wider range of clustering options (average, furthest and nearest neighbour). However, Mothur failed to process the huge, terabyte-sized, distance matrices derived from full MSAs of the extensive dataset [24], forcing us to reduce information content by including only unique sequences in the MSA. Regarding the results obtained with CROP, while proposing a new, promising and rigorous approach, it was unable to complete the analyses or produced anomalous results in several cases. This may be due to CROP being a new method that is still actively refined.

We have also considered variability in the results of the clustering algorithms. There are two sources of variability. One is due to the sub sampling required to build rarefaction curves and estimate actual diversity. The other source depends on algorithmic randomness, and after Studentized correction, the results from 20 repeated analyses show that ESPRIT consistently produces the same results, Mothur produces a slight variability, depending on the analysed dataset, and will not normally affect the diversity results, and CROP, which uses an MCMC step, has a higher but still low variability that should have a minimal impact on the results, although this may rarely lead to counter-intuitive diversity estimates as separate runs are used for each similarity level.

### Suitability for differential diversity studies

The results obtained from the analysis of the short-length hypervariable V6 16S rDNA sequences from soil were in all cases (with the exception of CROP for GTZ and glyphosate data) in good agreement with those previously reported for the same set of sequences, confirming that the reduction in species richness was more evident in the GTZ-treated soil at the first sampling time, and that the relative recovery from this herbicide treatment appeared to be poorer than that from the glyphosate treatment [32]. All of the workflows used, except CROP, were able to show differences in species richness among the different herbicide treatments used, and the untreated soil was the one that contained the highest bacterial diversity. This is in accordance with the results we have obtained from other field studies (data not shown), yet a general conclusion may not be reached just from our soil data.

### Workflow accuracy

Use of our reference set of 60 short-length hypervariable V6 16S rDNA sequences shows that MSAs obtained using MUSCLE and MAFFT produce the same results, regardless of the distance calculation method (with the exception of MAFFT), while the Mothur guided alignment underestimates diversity when dealing with a small number of OTUs. The method used for distance calculation also affects the number of OTUs, as MAFFT is unable to discriminate between different dissimilarity levels (even at 18%, data not shown), considering the small number of OTUs found. Jukes-Cantor or Mothur distances slightly underestimate the number of OTUs.

No aberrant combinations were found when nearly full-length 16S rDNA sequences were analysed, thus indicating that when dealing with long sequences, Mothur reference-guided alignments may be preferred. When dealing with short length sequences, the ESPRIT package and the RDP pipeline are the most accurate on the three dissimilarity levels. When analysing a large number of long sequences, computational time requirements adversely affect the use of the ESPRIT package due to the longer time needed, while the use of Mothur alone produces acceptable estimates in a reasonable time.

For even larger numbers of short length sequences in the range of hundreds of thousands [24], a filtering step that reduces sequence numbers is needed. ESPRIT and Otupipe automatically apply an initial filtering step and hence can directly work with these huge datasets, producing acceptable results. The RDP web-based pipeline may experience troubles or long delays with some datasets, but does generally give good results. In the case of MAFFT, Mothur and MUSCLE, the initial selection of unique sequences and optional preclustering steps helps by significantly reducing the data to manageable sizes.

In conclusion, the RDP web-based pipeline is most convenient for general use, but when dealing with very large or many datasets, or when timely results are needed, ESPRIT and Otupipe are very efficient and can be used locally to produce acceptable results in every circumstance. In all the cases tested, RDP and ESPRIT always rendered more accurate and similar results, although perhaps in specific circumstances, and depending on the number of sequences, length and variability, combination workflows may still be an attractive option.

## Methods

### Test datasets of 16S rDNA sequences

To check for accuracy, we constructed a reference data set with sequences retrieved from the NCBI and trimmed to obtain the V6 region (from positions 963 to 1063 in *E. coli*) using 60 sequences of different bacterial species from 59 different genera, according to NCBI taxonomy (accession numbers: AF538931, AF363135, DQ310706, AY167839, X90760, AM743175, AY367026, AF530131, U855746, EU360125, AM747393, FJ486138, AY691545, DQ366688, EF076758, AY234615, AM936268, AJ233945, FJ418118, AM935473, EF606819, AJ298940, EF173341, EF019646, CU466738, AM935145, AM935820, EF212893, AM59107, FM209153, EU375221, EF054879, EF466123, EF466120, EU722519, AF328681, EU58528, EU593733, AM935529, EU631792, AJ519368, AM935078, AY730501, EU630729, EU634621, DQ263467, X99390, DQ676361, AY852181, EU930421, DQ989484, AF448044, AJ224039, AB360346, AF114805, AY894325, EU551675, EU427317, AJ314848, AB037012). The accuracy of non-parametric estimators (ACE, Chao1) is expected to depend on changes in the ratio of OTUs to sequence numbers and OTU cluster size. To test their accuracy, the base dataset was copied 50 times to build a second set of 3000 sequences, and with the same number of OTUs.

To test the MSA step and richness estimators (ACE, Chao1), we simulated natural variability by generating two new 3000 sequence datasets mutating 50 copies of each sequence using EMBOSS [41] to introduce a single random mutation (indel or point mutation): one set had the 50 different mutants of each sequence stacked and the second one had them interspersed. Alignments were inspected using Seaview [42].

Recent studies tend to consider regions longer than V6 obtained from 454 sequencing. We obtained two datasets described by Quince [26], an artificial community pyrosequenced GSFLX dataset of 34308 V5 sequences with an average length of 266 nt and a maximum length of 289 nt, from 90 different clones and the Priest Pot pyrosequenced environmental DNA GSFLX data with 16222 sequences (both after initial noise removal) with an average length of 257 nt and a maximum length of 303 nt. These datasets were processed as above but in addition, we also calculated Mothur alignments with "trump" filtering to remove uneven ends.

To test the effect of nearly-full length sequences, two additional experimental data sets were used: one consisting of 1132 16S rDNA sequences from tall grass prairie soil samples with high diversity [37], with an average length of 1487 nt and a maximum length of 1542 nt, and a second one consisting of 1130 sequences from the axillary vault of patient HV5 of the human skin microbiome data with low diversity [23,38], with an average length of 1344 nt and a maximum length of 1387 nt, by retrieving the sequences from the EBI and assembling each dataset in a single file.

Recent advances in pyrosequencing technology have enabled Illumina reads to reach a length that is suitable for hypervariable region sequencing, yielding huge numbers of sequence reads. We have simulated this situation using the well-known dataset of Huse *et al*., [24] consisting of 340150 sequences (23445 of which are unique), with an average length of 96 nt and a maximum length of 169 nt.

To evaluate suitability for the study of differential environmental effects we have used a real-world experimental dataset consisting of short length sequences from rhizobacterial V6 16S rDNA obtained as previously described [32]. In synthesis, we collected soil samples from glyphosate-tolerant maize, event NK603 cultivars that had been treated in pre-emergence with the herbicide Harness^®^GTZ (GTZ henceforth) or in post-emergence with glyphosate (Roundup^®^Plus), and from untreated soil. Samples were pooled seven days after glyphosate application (first sampling time) and just before crop harvesting (final sampling time). Sequence counts obtained from the soils at the first sampling time were 3467 (untreated) with an average length of 103 nt and a maximum length of 243 nt, 5025 (glyphosate-treated) with an average length of 102 nt and a maximum length of 277 nt, and 3843 (GTZ-treated) with an average length of 99 nt and a maximum length of 269 nt, and at the final sampling time were 1814 with an average length of 101 nt and a maximum length of 157 nt, 1796 with an average length of 100 nt and a maximum length of 128 nt, and 1526 with an average length of 99 nt and a maximum length of 153 nt, respectively.

Additional file 3 contains the FASTA formatted sequences of all the datasets used in this study.

### Sequence processing

Differential soil data experimental sequences were filtered by 454 software, and additionally cleaned by eliminating those containing ambiguous base calls and sequences shorter than 50 nt after removing the primer sequence, as these account for 50% of all NGS errors [2]. We have omitted analysing the effect of different pre-processing strategies, as this was not within the scope of this study.

### Sequence analysis workflows

For the combined workflows the first step consists of obtaining the MSA. We test three different alignment approaches: a) Iterative progressive alignments by Log-Expectation comparison as implemented in MUSCLE version 3.7 [14] with parameters -diags -maxiters 4 -stable. b) A fast group-to-group alignment based on partition trees implemented in MAFFT version 6.712 [16] with parameters -ep 0.123 -retree 1 -nofft -parttree, and c) an initial 8-mer search followed by Needleman-Wunsch pairwise alignments against a reference alignment implemented in Mothur version 1.14.0, using the SILVA-98 [43] and Greengenes [44] reference alignment, although only SILVA guided results are reported because it has been proven more effective in aligning the 16S rRNA hypervariable regions [11]. For each dataset we generated two Mothur alignments: one with and one without a pre-clustering step, filtering the result to remove redundant gaps using the standard approach (default parameters) described in the Mothur documentation, and fixing gap characters for compatibility with other programs.

Using these four initial MSA, we calculate the distance matrices using three different approaches for each alignment: a) evolutionary distance estimation using Jukes-Cantor correction [34] for multiple substitution as implemented in DNADIST from the PHYLIP software package version 3.67 [33]; b) simple distance estimation counting gaps only once, penalizing terminal gap, with a cut-off value of 0.11 and increasing precision to 1000 using Mothur [19]; and c) an approximate 6-mer distance calculation method implemented in MAFFT version 6.712 [16]. The MAFFT output file was converted to a lower triangular matrix to produce the clustering input.

The cluster analysis comprises the generation of OTUs and calculation of species richness with the Chao1 and ACE estimators at three dissimilarity levels (3%, 5% and 10%). Clustering was performed using the three methods (average, nearest and farthest neighbour) available from Mothur [19], although we report only average neighbour calculations, as they are generally considered more accurate [11,25].

Beside these combined workflow procedures, we have included other methods that do not require an initial MSA: the ESPRIT software package [20], the recently published CROP package [23], the Otupipe pipeline (http://drive5.com/otupipe) and the RDP pyrosequencing pipeline [21]. ESPRIT uses an efficient k-tuple based distance filter before aligning sequences using the Needleman-Wunsch method [18] and computes pairwise distances using the "quickdist" algorithm [20] to perform complete-link hierarchical clustering; and CROP filters sequences first to reduce their number, then uses a modified Needleman-Wunsch alignment, performs a "quickdist" distance calculation, and finally an unsupervised Bayesian clustering method with MCMC sampling. Otupipe relies on UCHIME [45] to remove chimeras and on USEARCH to perform sequence comparisons and clustering [22]. RDP builds an initial MSA using the Infernal [36] tool and then directly proceeds to perform a complete linkage clustering using its own implementation.

To compare diversity estimation, we have compared rarefaction curves as well as ACE and Chao1 values obtained in all workflows involving Mothur and ESPRIT clustering. As CROP only produces OTU counts and composition, only these were considered in diversity estimate comparisons.

The results collected from all the different sources were processed to a common format (Mothur summary file format) and converted to tab-delimited data for further analysis and display using gnuplot.

### Assessment of clustering consistency

Clustering methods often resort to random decisions, which may lead to different results in different runs using the same initial data and conditions. We have analysed the consistency of the results obtained with different datasets: analysis of short sequences using our synthetic mutated datasets (60 species mutated 50 times, with consecutive or interspersed sequences) and long sequences using a 16S rRNA dataset from Youssef et al. These datasets were analysed using CROP, ESPRIT and Mothur (with and without pre-clustering, using the average neighbour distance clustering method), repeating the analysis 20 times, collecting the number of OTUs reported, and calculating mean, standard deviation and standard error of the mean to derive corrected 95% confidence intervals using Student's T value for N-1 degrees of freedom to account for the relatively small sample size.

## Authors' contributions

JB and JRV carried out the data collection and analysis, and drafted the manuscript. RPM conceived the study, and participated in its design and coordination and helped to draft the manuscript. All authors read and approved the final manuscript.

## Supplementary Material

Additional file 1 **Table S1.1 OTU counts**. J-C: Jukes-Cantor; 50 × 60 seq mut inter: 50 mutated copies of the 60 sequences dataset interleaved; 50 × 60 seq mut stack: 50 mutated copies of the 60 sequences dataset stacked; NC: not computable; t1, t2: samples taken at time 1 or time 2 (see Methods). Values marked with an * were computed using only unique sequences. Table S1.2 ACE estimates J-C: Jukes-Cantor; 50 × 60 seq mut inter: 50 mutated copies of the 60 sequences dataset interleaved; 50 × 60 seq mut stack: 50 mutated copies of the 60 sequences dataset stacked; NC: not computable; t1, t2: samples taken at time 1 or time 2 (see Methods). Values marked with an * were computed using only unique sequences. CROP, Outpipe and RDP does not compute the ACE estimator. Table S1.3 Chao1 estimates J-C: Jukes-Cantor; 50 × 60 seq mut inter: 50 mutated copies of the 60 sequences dataset interleaved; 50 × 60 seq mut stack: 50 mutated copies of the 60 sequences dataset stacked; NC: not computable; t1, t2: samples taken at time 1 or time 2 (see Methods). Values marked with an * were computed using only unique sequences. CROP and Outpipe does not compute the Chao1 estimator. Click here for file

Additional file 2 **Table S2. Computational time needed for each step in the diversity analysis**. J-C: Jukes-Cantor. Time is indicated as hours: minutes: seconds. *Alignment was calculated using -maxiter 2. Click here for file

Additional file 3 **This file contains the FASTA formatted sequences of all the datasets used in this study**. Zip file contents are briefly described in the included README file. Click here for file
