# Supplementary material for: Estimation of bacterial diversity using next generation sequencing of 16S rDNA: a comparison of different workflows
Source: BMC Bioinformatics. 2011 Dec 14;12:473. doi: 10.1186/1471-2105-12-473 (PMC3258296; doi:10.1186/1471-2105-12-473)
Supplement: Additional file 1 — Table S1.1 OTU counts. J-C: Jukes-Cantor; 50 × 60 seq mut inter: 50 mutated copies of the 60 sequences dataset interleaved; 50 × 60 seq mut stack: 50 mutated copies of the 60 sequences dataset stacked; NC: not computable; t1, t2: samples taken at time 1 or time 2 (see Methods). Values marked with an * were computed using only unique sequences. Table S1.2 ACE estimates J-C: Jukes-Cantor; 50 × 60 seq mut inter: 50 mutated copies of the 60 sequences dataset interleaved; 50 × 60 seq mut stack: 50 mutated copies of the 60 sequences dataset stacked; NC: not computable; t1, t2: samples taken at time 1 or time 2 (see Methods). Values marked with an * were computed using only unique sequences. CROP, Outpipe and RDP does not compute the ACE estimator. Table S1.3 Chao1 estimates J-C: Jukes-Cantor; 50 × 60 seq mut inter: 50 mutated copies of the 60 sequences dataset interleaved; 50 × 60 seq mut stack: 50 mutated copies of the 60 sequences dataset stacked; NC: not computable; t1, t2: samples taken at time 1 or time 2 (see Methods). Values marked with an * were computed using only unique sequences. CROP and Outpipe does not compute the Chao1 estimator. [file 1471-2105-12-473-S1.PDF]

Table S1.1 OTU counts

Alignment	% distance	CROP	ESPRIT	Mafft			Mothur			Mothur+preclustering			Muscle				
Distance		CROP	ESPRIT	J-C	Mafft	Mothur	J-C	Mafft	Mothur	J-C	Mafft	Mothur	J-C	Mafft	Mothur	Otupipe	RDP
Clustering		CROP	ESPRIT		Mothur			Mothur			Mothur			Mothur			
60 sequences	0.03	59	59	58	59	59	55	41	48	44	47	48	58	59	59	59	59
	0.05	59	57	56	59	56	55	40	48	44	47	48	56	59	56	57	57
	0.10	59	52	50	59	51	55	36	48	44	47	48	50	59	55	52	52
50 x 60 sequences	0.03	60	59	58	59	59	2992	252	48	43	47	48	63	59	66	59	59
	0.05	60	57	57	59	57	2992	251	48	43	47	48	59	59	62	57	57
	0.10	60	52	51	59	54	2992	243	48	43	47	48	59	59	56	52	52
50 x 60 seq mut inter	0.03	1959	193	141	2289	261	2947	899	1087	1198	1328	1080	70	2287	264	139	59
	0.05	1954	59	101	1708	119	2947	685	923	1198	1328	938	59	1707	64	91	58
	0.10	1955	56	85	80	96	2947	477	736	1198	1328	938	44	80	57	59	53
50 x 60 seq mut stack	0.03	1865	205	132	2289	279	2985	2999	1100	1333	1346	1087	2999	2288	571	144	59
	0.05	1850	59	113	1777	121	2985	2999	930	1333	1346	940	2999	1785	466	95	57
	0.10	1850	56	113	1270	121	2985	2999	888	1333	1346	915	2999	1269	466	61	52
Artificial community	0.03	41	248	686	31933	1756	49	4276	113	61	3864	136	146	33491	258	66	250
	0.05	25	77	686	31933	1756	36	4276	53	40	3141	65	146	33491	258	39	94
	0.10	15	38	686	31933	1756	33	4276	53	36	3141	46	146	33491	258	24	43
Priest Pot soil	0.03	562	1115	3764	15984	6672	640	2824	766	662	1905	810	4059	15718	6433	793	1209
	0.05	246	773	3764	15984	6672	537	2824	642	482	1625	575	4059	15718	6433	570	862
	0.10	42	394	3764	15984	6672	537	2824	642	482	1625	575	4059	15718	6433	302	456
Axillary skin	0.03	1009	59	47	266	50	47	269	54	47	287	55	50	266	58	49	60
	0.05	1009	43	39	126	39	39	126	40	39	127	40	39	126	40	37	43
	0.10	1009	26	39	109	37	36	109	38	36	109	38	37	109	38	26	27
Prairie soil	0.03	1128	504	490	1007	503	491	977	535	491	977	536	480	1007	487	490	504
	0.05	1128	340	346	895	351	352	872	396	353	877	397	337	895	339	336	348
	0.10	1128	162	183	714	181	184	689	204	268	836	305	175	714	173	160	175
Control-t1	0.03	812	1631	1936	2792	2112	3464	2789	1730	2479	2471	2063	1762	2790	2278	1314	1762
	0.05	176	1225	1633	2541	1814	3464	2544	1342	2479	2471	2063	1482	2538	1856	986	1335
	0.10	38	661	1101	1868	1244	3464	1868	832	2479	2471	2063	950	1871	1347	522	779
Control-t2	0.03	286	1053	1087	1588	1215	1622	1589	1128	1460	1459	1143	1247	1587	1352	881	1106
	0.05	100	813	920	1503	1047	1622	1503	911	1460	1459	937	1134	1501	1236	696	877
	0.10	28	477	631	1185	716	1622	1181	565	1460	1459	937	847	1181	1002	390	547
GTZ-t1	0.03	414	1227	1025	1971	1655	3842	1969	1310	1958	1961	1890	1257	1970	1800	938	1236
	0.05	117	916	830	1825	1337	3842	1826	1030	1958	1961	1890	1042	1825	1488	717	926
	0.10	29	495	505	1325	854	3842	1323	624	1958	1961	1890	647	1322	1021	402	579
GTZ-t2	0.03	625	922	975	1329	1163	823	1328	982	826	1252	1020	950	1330	1059	795	901
	0.05	228	730	865	1273	1011	687	1272	820	826	1252	1020	827	1274	920	633	738
	0.10	20	459	606	1004	745	441	1004	526	826	1252	1020	616	1006	717	385	498
Glyphosate-t1	0.03	454	1951	1977	3296	2646	5024	3323	2122	3134	3135	2516	2020	3296	2772	1428	1932
	0.05	172	1355	1618	2956	2078	5024	2987	1641	3134	3135	2516	1681	2965	2177	1059	1409
	0.10	34	713	1037	2095	1338	5024	2128	953	3134	3135	2516	1115	2097	1575	588	830
Glyphosate-t2	0.03	477	1102	1088	1577	1326	1090	1574	1209	1498	1495	1221	1238	1577	1477	948	1094
	0.05	191	861	912	1489	1104	1090	1490	1007	1498	1495	1221	1040	1490	1280	739	872
	0.10	16	481	599	1196	740	1090	1198	643	1498	1495	1221	689	1198	992	430	551
Huse *et al.*	0.03	NC	6464	23442*	23444*	18436*	23441*	23441*	18210*	31524	15601	14776	23443*	23444*	21318*	2149	4228
	0.05	NC	3308	23442*	23444*	18436*	23441*	23441*	18210*	31524	15601	14776	23443*	23444*	21318*	1422	2932
	0.10	NC	1402	23442*	23444*	18436*	23441*	23441*	18210*	31524	15601	14776	23443*	23444*	21318*	878	1777

Legend. J-C: Jukes-Cantor; 50 x 60 seq mut inter: 50 mutated copies of the 60 sequences dataset interleaved; 50 x 60 seq mut stack: 50 mutated copies of the 60 sequences dataset stacked; NC: not computable; t1, t2: samples taken at time 1 or time 2 (see Methods). Values marked with an * were computed using only unique sequences.

Table S1.2 ACE estimates

Alignment		CROP	ESPRIT	Mafft			Mothur			Mothur+preclustering			Muscle				
**Distance**	**% distance**	**CROP**	**ESPRIT**	**J-C**	**Mafft**	**Mothur**	**J-C**	**Mafft**	**Mothur**	**J-C**	**Mafft**	**Mothur**	**J-C**	**Mafft**	**Mothur**	**Outpipe**	**RDP**
**Clustering**		**CROP**	**ESPRIT**	**Mothur**			**Mothur**			**Mothur**			**Mothur**				
**60 sequences**	0.03	NC	1770.00	870.00	870.00	1770.00	330.00	337.49	72.35	264.00	1128.00	NC	870.00	1770.00	1770.00	NC	NC
	0.05	NC	570.00	420.00	870.00	420.00	330.00	337.49	83.25	264.00	1128.00	NC	420.00	1770.00	420.00	NC	NC
	0.10	NC	218.37	184.86	870.00	182.57	330.00	337.49	217.20	264.00	1128.00	NC	184.86	1770.00	330.00	NC	NC
**50 x 60 sequences**	0.03	NC	NC	NC	NC	NC	561000.00	NC	NC	206.40	1128.00	NC	73.00	NC	87.00	NC	NC
	0.05	NC	NC	NC	NC	NC	561000.00	NC	NC	206.40	1128.00	NC	NC	NC	72.00	NC	NC
	0.10	NC	NC	NC	NC	NC	561000.00	NC	NC	206.40	1128.00	NC	NC	NC	NC	NC	NC
**50 x 60 seq mut inter**	0.03	NC	234.34	218.55	59963.07	202.16	83405.66	9195.27	7494.49	6076.88	882456.00	16331.23	125.00	58445.48	468.79	NC	NC
	0.05	NC	NC	111.19	5004.91	118.68	83405.66	7349.76	5065.03	6076.88	882456.00	11322.44	0.00	4849.28	0.00	NC	NC
	0.10	NC	NC	91.97	84.00	106.35	83405.66	5093.66	3136.53	6076.88	882456.00	11322.44	0.00	84.53	0.00	NC	NC
**50 x 60 seq mut stack**	0.03	NC	248.90	224.42	49160.29	567.79	298500.00	4498500.00	7069.40	64126.82	906531.00	19390.13	4498500.00	50553.89	1523.00	NC	NC
	0.05	NC	NC	175.32	6561.82	151.50	298500.00	4498500.00	5302.32	64126.82	906531.00	12532.05	4498500.00	6484.62	1529.44	NC	NC
	0.10	NC	NC	175.32	2088.16	151.50	298500.00	4498500.00	4725.96	64126.82	906531.00	11725.16	4498500.00	2120.15	1529.44	NC	NC
**Artificial community**	0.03	NC	303.93	2705.55	230643.66	6744.42	116.00	7330.98	830.41	127.24	52657.97	944.09	465.70	703188.02	1168.00	NC	NC
	0.05	NC	129.82	2705.55	230643.66	6744.42	72.80	7330.98	176.64	78.44	15958.09	341.47	465.70	703188.02	1168.00	NC	NC
	0.10	NC	41.23	2705.55	230643.66	6744.42	58.18	7330.98	176.64	66.39	15958.09	106.36	465.70	703188.02	1168.00	NC	NC
**Priest Pot soil**	0.03	NC	2233.30	14670.51	544732.03	44712.85	1813.58	10338.74	2512.18	2502.58	25291.77	3174.10	17616.08	252953.77	40232.64	NC	NC
	0.05	NC	1363.31	14670.51	544732.03	44712.85	1278.72	10338.74	1763.41	1317.21	12535.70	1847.09	17616.08	252953.77	40232.64	NC	NC
	0.10	NC	489.71	14670.51	544732.03	44712.85	1278.72	10338.74	1763.41	1317.21	12535.70	1847.09	17616.08	252953.77	40232.64	NC	NC
**Axillary skin**	0.03	NC	78.54	65.38	3122.76	69.24	65.12	2781.96	68.55	65.09	3466.55	71.09	68.27	3209.05	83.09	NC	NC
	0.05	NC	61.44	63.20	364.29	63.20	63.20	298.49	67.84	54.67	322.48	57.65	63.20	364.29	67.84	NC	NC
	0.10	NC	35.78	63.20	265.65	61.20	50.36	217.38	47.97	49.76	254.82	49.35	61.20	265.65	62.20	NC	NC
**Prairie soil**	0.03	NC	1324.36	2106.14	9435.18	2271.70	2152.00	13182.37	2315.61	2415.35	8246.54	2566.71	2086.27	9435.18	2092.53	NC	NC
	0.05	NC	648.37	1046.71	10404.71	1053.74	1040.95	10526.40	1471.03	1034.93	9678.32	1453.09	988.91	10404.71	963.32	NC	NC
	0.10	NC	241.95	453.49	4256.97	463.93	382.23	4139.62	475.43	763.25	8176.14	1010.77	390.67	4256.97	393.03	NC	NC
**Control-t1**	0.03	NC	3361.68	8782.73	26570.62	11932.68	2001614.67	26510.72	8292.07	1537599.75	306527.55	6949.23	6523.74	24986.71	17448.44	NC	NC
	0.05	NC	2070.28	6413.95	15702.57	8326.35	2001614.67	15183.64	5624.86	1537599.75	306527.55	6949.23	4506.43	15268.12	9755.89	NC	NC
	0.10	NC	812.26	3589.29	8354.00	4255.51	2001614.67	8494.76	3778.61	1537599.75	306527.55	6949.23	2126.17	8696.34	4570.69	NC	NC
**Control-t2**	0.03	NC	2848.94	5781.12	22204.90	9321.28	7662.26	20471.22	5315.00	1066530.00	532899.75	8167.67	10648.39	19512.70	14911.50	NC	NC
	0.05	NC	1570.92	4176.36	17940.29	5081.02	7662.26	16956.49	3561.98	1066530.00	532899.75	4965.48	7589.93	16472.42	10808.49	NC	NC
	0.10	NC	630.17	2154.08	8081.14	3141.96	7662.26	7780.64	1921.12	1066530.00	532899.75	4965.48	3199.61	7815.92	6357.20	NC	NC
**GTZ-t1**	0.03	NC	2858.19	3435.01	21101.48	12541.69	7382403.00	22094.92	6761.57	480199.50	1923741.00	89494.97	5524.14	24113.74	17688.26	NC	NC
	0.05	NC	1597.03	2740.76	12314.06	7756.28	7382403.00	11962.29	3967.09	480199.50	1923741.00	89494.97	3650.85	13321.37	9903.85	NC	NC
	0.10	NC	631.22	1550.36	5290.68	2609.17	7382403.00	5302.66	2524.68	480199.50	1923741.00	89494.97	1561.66	5338.39	3890.21	NC	NC
**GTZ-t2**	0.03	NC	2618.68	5898.55	9292.36	10428.44	4441.78	9591.27	7733.66	3829.47	784378.00	4653.94	4500.14	9459.68	6979.54	NC	NC
	0.05	NC	1662.71	4451.76	7611.84	6490.42	3238.90	13278.76	5355.80	3829.47	784378.00	4653.94	3191.69	7886.02	4698.09	NC	NC
	0.10	NC	679.65	2024.98	6950.17	3065.70	1382.49	6791.48	2005.98	3829.47	784378.00	4653.94	1521.67	6740.18	2405.74	NC	NC
**Glyphosate-t1**	0.03	NC	4225.48	8673.84	29200.13	18599.60	12622800.00	28809.05	11649.55	2457056.00	4915680.00	11216.26	8189.54	27205.00	19319.33	NC	NC
	0.05	NC	2245.89	5994.16	19460.38	10525.79	12622800.00	20400.29	7908.56	2457056.00	4915680.00	11216.26	6009.02	20412.66	11816.47	NC	NC
	0.10	NC	883.74	2868.13	10232.77	4610.74	12622800.00	10334.50	3264.22	2457056.00	4915680.00	11216.26	2493.84	9728.76	6054.95	NC	NC
**Glyphosate-t2**	0.03	NC	3211.61	7608.78	27105.54	14418.44	1386.43	31385.49	10683.96	1122751.00	280125.63	9862.69	8469.13	30220.06	20848.07	NC	NC
	0.05	NC	1787.99	4884.83	20443.86	8640.75	1386.43	16951.78	6500.70	1122751.00	280125.63	9862.69	5108.37	17072.38	10464.76	NC	NC
	0.10	NC	627.23	1984.03	7860.39	3095.81	1386.43	7533.31	2880.51	1122751.00	280125.63	9862.69	1767.23	8256.99	5194.24	NC	NC
**Huse *et al.***	0.03	NC	10398.89	91599614.99*	274822289.99*	45928.38*	68696780.62*	274822289.99*	44072.82*	12166438.8	40573000.7	159671.27	425215.51*	274822289.99*	139129.96*	NC	NC
	0.05	NC	5900.45	91599614.99*	274822289.99*	45928.38*	68696780.62*	274822289.99*	44072.82*	12166438.8	40573000.7	159671.27	425215.51*	274822289.99*	139129.96*	NC	NC
	0.10	NC	2405.29	91599614.99*	274822289.99*	45928.38*	68696780.62*	274822289.99*	44072.82*	12166438.8	40573000.7	159671.27	425215.51*	274822289.99*	139129.96*	NC	NC

Legend. J-C: Jukes-Cantor; 50 x 60 seq mut inter: 50 mutated copies of the 60 sequences dataset interleaved; 50 x 60 seq mut stack: 50 mutated copies of the 60 sequences dataset stacked; NC: not computable; t1, t2: samples taken at time 1 or time 2 (see Methods). Values marked with an * were computed using only unique sequences. CROP, Outpipe and RDP does not compute the ACE estimator.

Table S1.3 CHAO1 estimates

Alignment	% distance	CROP	ESPRIT	Mafft			Mothur			Mothur+preclustering			Muscle				
**Distance**		CROP	ESPRIT	J-C	Mafft	Mothur	J-C	Mafft	Mothur	J-C	Mafft	Mothur	J-C	Mafft	Mothur	Outpipe	RDP
**Clustering**		CROP	ESPRIT		Mothur			Mothur			Mothur			Mothur			
	0.03	NC	885.50	571.33	885.50	885.50	259.17	66.00	220.20	200.00	564.50	1176.00	571.33	885.50	885.50	NC	885.50
**60 sequences**	0.05	NC	414.75	321.20	885.50	321.20	259.17	67.08	220.20	200.00	564.50	1176.00	321.20	885.50	321.20	NC	414.75
	0.10	NC	193.43	173.00	885.50	163.87	259.17	73.50	220.20	200.00	564.50	1176.00	173.00	885.50	259.17	NC	193.42
	0.03	NC	59.00	58.00	59.00	59.00	497507.11	20152.00	48.00	160.17	564.50	1176.00	66.00	59.00	73.50	NC	885.50
**50 x 60 sequences**	0.05	NC	57.00	57.00	59.00	57.00	497507.11	20151.00	48.00	160.17	564.50	1176.00	59.00	59.00	65.00	NC	414.75
	0.10	NC	52.00	51.00	59.00	54.00	497507.11	20143.00	48.00	160.17	564.50	1176.00	59.00	59.00	57.00	NC	193.42
	0.03	NC	215.94	184.94	12600.51	370.44	80468.69	3418.03	3084.50	5506.42	441228.50	1080.00	92.50	12535.96	445.79	NC	885.50
**50 x 60 seq mut inter**	0.05	NC	59.00	110.75	4286.33	130.33	80468.69	2762.06	2355.13	5506.42	441228.50	938.00	59.00	4334.91	74.00	NC	571.33
	0.10	NC	56.00	91.00	82.10	109.00	80468.69	1905.55	1742.23	5506.42	441228.50	938.00	44.00	82.10	57.00	NC	233.16
	0.03	NC	232.72	190.58	11362.16	411.47	278545.31	2249250.50	3044.61	59281.07	453266.00	6439.56	2249250.50	11310.85	956.39	NC	885.50
**50 x 60 seq mut stack**	0.05	NC	59.00	152.00	5108.78	150.55	278545.31	2249250.50	2565.08	59281.07	453266.00	4740.67	2249250.50	5089.90	893.00	NC	414.75
	0.10	NC	56.00	152.00	2059.39	150.55	278545.31	2249250.50	2267.59	59281.07	453266.00	4375.75	2249250.50	2067.85	893.00	NC	193.42
	0.03	NC	296.62	1648.77	215781.02	3894.70	58.00	6870.39	260.50	76.30	25767.15	452.00	279.00	686032.32	643.45	NC	327.65
**Artificial community**	0.05	NC	100.33	1648.77	215781.02	3894.70	41.25	6870.39	75.67	51.25	8367.25	132.67	279.00	686032.32	643.45	NC	111.50
	0.10	NC	38.43	1648.77	215781.02	3894.70	36.75	6870.39	75.67	45.00	8367.25	64.20	279.00	686032.32	643.45	NC	47.20
	0.03	NC	1962.61	8606.00	534646.70	21226.20	1261.10	6598.95	1624.10	1520.77	10984.72	1892.42	9764.26	244876.99	19750.37	NC	2221.94
**Priest Pot soil**	0.05	NC	1287.09	8606.00	534646.70	21226.20	925.57	6598.95	1245.80	855.82	6158.31	1126.67	9764.26	244876.99	19750.37	NC	1426.78
	0.10	NC	488.94	8606.00	534646.70	21226.20	925.57	6598.95	1245.80	855.82	6158.31	1126.67	9764.26	244876.99	19750.37	NC	582.53
	0.03	NC	90.67	74.20	1110.60	80.60	74.20	1166.00	81.20	74.20	1256.23	85.60	80.60	1193.00	96.50	NC	84.42
**Axillary skin**	0.05	NC	60.50	54.17	249.47	54.17	54.17	249.47	57.50	54.17	250.47	57.50	54.17	249.47	57.50	NC	54.14
	0.10	NC	33.00	54.17	223.23	52.17	49.00	223.23	46.00	49.00	244.00	48.00	52.17	223.23	53.17	NC	36.00
	0.03	NC	1244.53	1336.05	8058.28	1389.62	1371.89	6646.13	1467.79	1429.00	6834.03	1527.11	1245.69	8058.28	1312.44	NC	1162.71
**Prairie soil**	0.05	NC	651.78	791.72	4456.32	840.27	814.47	4057.52	1065.40	824.38	4066.67	1063.12	727.02	4456.32	733.06	NC	675.30
	0.10	NC	250.44	326.04	2296.82	351.63	321.31	2128.82	378.14	570.03	3807.08	741.41	307.84	2296.82	305.84	NC	283.37
	0.03	NC	3328.63	5161.93	14088.10	6767.20	1500346.50	14146.25	5305.37	1024654.33	277655.55	6927.95	4137.34	13812.61	9342.46	NC	4080.32
**Control-t1**	0.05	NC	2108.20	4070.91	8857.60	5037.26	1500346.50	8841.34	3529.52	1024654.33	277655.55	6927.95	3058.93	8874.72	5276.04	NC	2447.07
	0.10	NC	777.82	2447.65	4903.77	2900.81	1500346.50	4902.29	2089.85	1024654.33	277655.55	6927.95	1591.09	4902.42	3050.37	NC	1068.10
	0.03	NC	2624.56	3077.06	11208.01	4269.83	6915.96	10839.71	4473.05	533265.50	355024.33	4963.01	4736.51	10966.69	6459.50	NC	3079.23
**Control-t2**	0.05	NC	1492.68	2361.16	7970.06	2942.23	6915.96	7865.28	2982.14	533265.50	355024.33	2863.38	3823.86	7824.65	4965.51	NC	1733.64
	0.10	NC	626.23	1286.59	4577.09	1765.67	6915.96	4536.93	1549.55	533265.50	355024.33	2863.38	1905.48	4518.28	3376.14	NC	792.83
	0.03	NC	2627.84	2161.63	9705.44	6373.41	3691202.00	9770.33	4041.35	383574.20	961871.00	39405.62	3135.06	9957.12	9385.01	NC	2675.83
**GTZ-t1**	0.05	NC	1534.82	1776.41	7204.19	4059.58	3691202.00	7212.36	2626.81	383574.20	961871.00	39405.62	2257.74	7400.17	5349.16	NC	1556.23
	0.10	NC	666.59	1139.92	3475.22	1861.54	3691202.00	3464.12	1581.51	383574.20	961871.00	39405.62	1131.29	3499.22	2457.03	NC	800.00
	0.03	NC	2355.96	3080.19	8810.26	5306.88	2352.34	9199.80	3787.84	2473.25	392189.50	4603.60	2424.07	8861.28	3785.80	NC	2513.71
**GTZ-t2**	0.05	NC	1476.14	2420.01	7075.22	3578.51	1788.61	7351.33	2543.68	2473.25	392189.50	4603.60	1941.15	7172.37	2585.00	NC	1594.77
	0.10	NC	648.01	1208.06	3497.24	1842.85	882.09	3383.16	1184.13	2473.25	392189.50	4603.60	1149.21	3373.01	1479.37	NC	759.36
	0.03	NC	3999.43	4914.09	14358.21	8836.90	6311400.50	14714.20	6489.13	1637516.00	2457840.50	10683.78	4996.78	14499.70	11037.99	NC	3871.48
**Glyphosate-t1**	0.05	NC	2234.82	3648.11	10238.08	5954.47	6311400.50	10391.12	4591.57	1637516.00	2457840.50	10683.78	3852.41	10250.21	6818.06	NC	2545.47
	0.10	NC	865.64	1907.94	5617.75	2841.78	6311400.50	5777.56	2000.22	1637516.00	2457840.50	10683.78	1853.23	5513.90	3678.37	NC	1137.86
	0.03	NC	2949.22	3798.62	12528.61	7240.50	1194.01	12714.65	5515.12	561376.00	223654.00	6057.59	4933.76	12513.43	10752.11	NC	3319.00
**Glyphosate-t2**	0.05	NC	1722.67	2721.58	9181.22	4292.80	1194.01	8971.92	3510.42	561376.00	223654.00	6057.59	2937.80	9416.00	5667.51	NC	1786.25
	0.10	NC	613.34	1317.11	4216.17	1997.71	1194.01	4165.31	1900.00	561376.00	223654.00	6057.59	1321.13	4184.21	3179.78	NC	835.87
	0.03	NC	10568.15	68693852.25*	137411145.50*	43626.47*	54950394.20*	137411145.50*	42970.44*	11054024.5	30425851.8	157282.19	413483.28*	137411145.50*	137427.74*	NC	8246.86
**Huse *et al.***	0.05	NC	5821.93	68693852.25*	137411145.50*	43626.47*	54950394.20*	137411145.50*	42970.44*	11054024.5	30425851.8	157282.19	413483.28*	137411145.50*	137427.74*	NC	6246.80
	0.10	NC	2324.36	68693852.25*	137411145.50*	43626.47*	54950394.20*	137411145.50*	42970.44*	11054024.5	30425851.8	157282.19	413483.28*	137411145.50*	137427.74*	NC	3056.91

Legend. J-C: Jukes-Cantor; 50 x 60 seq mut inter: 50 mutated copies of the 60 sequences dataset interleaved; 50 x 60 seq mut stack: 50 mutated copies of the 60 sequences dataset stacked; NC: not computable; t1, t2: samples taken at time 1 or time 2 (see Methods). Values marked with an * were computed using only unique sequences. CROP and Outpipe does not compute the Chao1 estimator.
