# Supplementary material for: Estimation of bacterial diversity using next generation sequencing of 16S rDNA: a comparison of different workflows
Source: BMC Bioinformatics. 2011 Dec 14;12:473. doi: 10.1186/1471-2105-12-473 (PMC3258296; doi:10.1186/1471-2105-12-473)
Supplement: Additional file 2 — Table S2. Computational time needed for each step in the diversity analysis. J-C: Jukes-Cantor. Time is indicated as hours: minutes: seconds. *Alignment was calculated using -maxiter 2. [file 1471-2105-12-473-S2.PDF]

Table S2. Computational time needed for each step in the diversity analysis

		Huse *et al*.	PriestPot	GTZ-t2	Prairie Soil
**CROP**	CROP	06:12:26	00:56:38	00:23:19	04:07:08
**Otupipe**	Otupipe	00:01:10	00:01:58	00:00:43	00:01:01
**ESPRIT**	ESPRIT	01:21:08	02:28:58	00:00:31	05:51:59
**Alignment**	MAFFT	00:04:19	00:04:19	00:03:55	00:04:18
	Mothur	00:47:22	00:44:47	00:28:50	00:10:14
	MUSCLE	01:09:06*	00:52:49*	00:07:10	00:52:41
**Distances**	JC	01:27:59	00:25:05	00:01:02	00:00:56
	MAFFT	03:20:20	00:34:00	00:02:36	00:02:35
	Mothur	01:14:38	00:25:28	00:00:07	00:00:21
**Clustering**	Mothur	00:10:15	00:09:49	00:00:05	00:00:15

Legend. J-C: Jukes-Cantor. Time is indicated as hours: minutes: seconds.
*Alignment was calculated using -maxiter 2.
